# *Fusobacterium nucleatum*-derived small extracellular vesicles facilitate tumor growth and metastasis via TLR4 in breast cancer

**DOI:** 10.1186/s12885-023-10844-z

**Published:** 2023-05-23

**Authors:** Guiqiu Li, Yan Sun, Yu Huang, Jie Lian, Shaoyuan Wu, Dixian Luo, Hui Gong

**Affiliations:** 1 Clinical Laboratory, Huazhong University of Science and Technology Union Shenzhen Hospital, Affiliated Shenzhen Sixth Hospital of Shenzhen University, No. 89 Taoyuan Road, Nanshan District, Shenzhen, 518052 PR China; 2 Shenzhen Nanshan District Maternal and Child Health Hospital, Shenzhen, 518052 PR China

**Keywords:** *Fusobacterium nucleatum*, Extracellular vesicles, Breast cancer, TLR4

## Abstract

**Background:**

The contributive role of the microbiome in tumor progression has been reported in multiple studies, such as the *Fusobacterium nucleatum* (*F. nucleatum*) in breast cancer (BC). This study aimed to explore the role of *F. nucleatum*-derived small extracellular vesicles (Fn-EVs) in BC and preliminarily uncover the mechanism.

**Methods:**

Ten normal and 20 cancerous breast tissues were harvested to investigate the gDNA expression of *F. nucleatum* and its relation with the clinical characteristics of BC patients. After isolating Fn-EVs by ultracentrifugation from *F. nucleatum* (ATCC 25,586), both MDA-MB-231 and MCF-7 cells were treated with PBS, Fn, or Fn-EVs, followed by being subjected to CCK-8, Edu staining, wound healing, and Transwell assays to detect their cell viability, proliferation, migration, and invasion. TLR4 expression in BC cells with diverse treatments was assessed by western blot. In vivo experiments were performed to verify its role in tumor growth and liver metastasis.

**Results:**

The *F. nucleatum* gDNA levels of breast tissues in BC patients were significantly higher than those in normal subjects, and positively associated with tumor size and metastasis. Fn-EVs administration significantly enhanced the cell viability, proliferation, migration, and invasion of BC cells, while knocking down TLR4 in BC cells could block these effects. Furthermore, in vivo study verified the contributive role of Fn-EVs in tumor growth and metastasis of BC, which might rely on its regulation of TLR4.

**Conclusions:**

Collectively, our results suggest that *F. nucleatum* plays an important role in BC tumor growth and metastasis by regulating TLR4 through Fn-EVs. Thus, a better understanding of this process may aid in the development of novel therapeutic agents.

**Supplementary Information:**

The online version contains supplementary material available at 10.1186/s12885-023-10844-z.

## Background

As the most commonly diagnosed cancer worldwide, breast cancer (BC) in China is accounting for 18% of the global BC cases in 2020, with approximately 0.42 million new cases [1]. However, the etiology of BC remains not completely uncovered and causal pathways are still hard to delineate. Due to increased body weight and continued decline in the fertility rate, the incidence rate of BC is increasing globally per year by an estimated 0.5% [2]. As reported, BC new cases number increased from 0.3 million in 2015 to 0.42 million in 2020 in China [1]. It is estimated that 3.2 million new cases of female BC by the year 2050 [3]. These alarming data manifest the urgent need for accurate and efficient screening and management of BC, and further advancing our knowledge of all possible factors contributing to the occurrence and progression of BC.

In the past few decades, the microbiota is considered essential for our physiology to function properly, mainly due to their production of metabolites [4]. There is a perfect balance between the microbiome and our body in a healthy state, which is known as symbiosis. When this symbiosis relationship is disrupted, microbial imbalances develop, which may lead to the development of multiple malignancies [5], including colorectal [6], lung [7], and stomach [8] cancers. Actually, accumulating studies indicated that the microbiome plays a role in more than 18% of malignancies and therapeutic resistance [9]. Previous studies have revealed that an overabundance of *Escherichia coli* could be observed in CRC tumorous biopsies in stage I to IV patients [10], while *Fusobacterium nucleatum* (*F. nucleatum*) was only observed in stage IV cancers [11]. Besides, the infection of both *Helicobacter pylori* [12] and *Mycobacterium tuberculosis* [13] have been implicated in the occurrence and progression of lung cancer.

For BC, multiple reports have provided evidence that breast tissue has its unique microbiome, which reflects its involvement in BC occurrence and progression [14]. Research by Hieken et al. uncovered the significantly different microbiota composition in the breast tissue between women with benign and malignant tumors [15]. Several microorganisms have been identified as the “tumor promoter” in BC. For instance, the tumoral overabundance of *Staphylococcus* and *Brevundimonas* has been found in primary breast tumors from patients with distant metastasis, which suggested that these microorganisms might be involved in the modulation of BC metastasis [16]. *F. nucleatum* has been considered the risk factor for various malignancies [17], including BC. A recent study demonstrated that *F. nucleatum* is capable of facilitating tumor growth and metastasis of BC, which could be a promising target for BC treatment [18]. However, its carcinogenesis mechanisms in BC have yet to be fully explored.

It’s widely accepted that the continuous growing, spreading, and metastasis of tumor cells depend on intercellular communication within cells, which could be mediated by the secretion of small extracellular vesicles (EVs) within a microenvironment [19]. EVs are nanosized membrane-encapsulated cell fragments shed from different domains of life, including bacteria, which have been proven to regulate diverse biological processes [20, 21]. As mentioned earlier, bacterial EVs involve interactions between bacteria and host cells [22]. For example, *Akkermansia muciniphila*-derived EVs suppress the production of IL-6 during colitis [23]. The administration of bacterial EVs derived from *S. enterica*, *S. aureus*, and *L. acidophilus* lead to the activation of anti-tumor immune responses and up-regulation of tumor-suppressor genes in tumor tissues [24]. Given that EVs produced by diverse cells or microorganisms have been reported to participate in the development of different types of tumors [25, 26], we wondered whether *F. nucleatum*-derived EVs (Fn-EVs) could facilitate the malignant behaviors of BC. Hence, the present study aimed to explore the role of Fn-EVs in BC and preliminarily uncover the mechanism.

## Methods

### Clinical samples collection

The study protocols were approved by the Ethics Committees of Huazhong University of Science and Technology Union Shenzhen Hospital and the Affiliated Shenzhen Sixth Hospital of Shenzhen University and performed in line with the provisions of the Helsinki Declaration of 1975. After being obtained written informed consent from the patients according to institutional guidelines, a total of 30 fresh breast tissues from 20 patients with BC and 10 patients with hyperplasia of the mammary glands were harvested during breast surgery. None of the subjects had received probiotic or antibiotic administration for at least three months before the sample collection.

### ***F. nucleatum*** detection

To assess the amounts of *F. nucleatum*, gDNA extracted from breast tissues of patients with BC or hyperplasia of the mammary glands was subjected to qPCR to test the Bacterial 16 S rRNA genes by using PGT as a reference gene [27]. The primers sequences used to detect *F. nucleatum* and PGT were listed as followed: forward *F. nucleatum*, CAACCATTACTTTAACTCTACCATGTTCA, reverse *F. nucleatum*, GTTGACTTTACAGAAGGAGATTATGTAAAAATC; forward PGT, ATCCCCAAAGCACCTGGTTT, reverse PGT, AGAGGCCAAGATAGTCCTGGTAA. *F. nucleatum* abundance in tumor versus normal breast tissue was calculated based on the 2^−ΔΔCt^ method [28]. Reaction conditions of amplification and detection were referred to in a previous study described by Castellarin, Warren, Freeman, Dreolini, Krzywinski, Strauss, Barnes, Watson, Allen-Vercoe, Moore and Holt [27].

### ***F. nucleatum*** culture and conditioned media collection

*F. nucleatum* strain ATCC 25,586 was grown in BD™ Columbia agar containing defibrinated sheep blood (5%) and cultured in an anaerobic chamber at 37 °C.

*F. nucleatum* conditioned media were collected once in two days, followed by sterile filtration using a 0.22-µm syringe filter. The filtered media was stored at -80 °C before EVs isolation.

### EVs isolation and characterization

EVs isolation was performed as an ultracentrifugation protocol reported in a recent study [29]. In brief, to obtain the *F. nucleatum*-derived EVs (Fn-EVs), the collected *F. nucleatum*-conditioned media were ultracentrifuged at 200,000× g for 60 min. Next, the obtained pellets were subjected to resuspension with PBS and filtered by a 0.22-µm syringe filter to finally obtain pure Fn-EVs.

The characterization of Fn-EVs, which included their morphology, number, size distribution, and protein concentration was performed using a transmission electron microscope (TEM), nanoparticle tracking analysis (NTA) with Nano-ZS 90 dynamic light scattering, and bicinchoninic acid (BCA) assay.

### Cell culture and treatment

Two BC cell lines (MDA-MB-231 and MCF-7) supplied by ATCC were grown in DMEM media with 10% FBS at 37 °C. To investigate the effect of Fn-EVs on BC cells, cells were divided into three groups: control, Fn, and Fn-EVs groups. After cell confluence reached approximately 85%, cells were given diverse treatments according to different groups (control: PBS; Fn: *F. nucleatum* suspension (1⋅10^9^ CFU/mL); Fn-EVs: 10 µg Fn-EVs.) and further incubated for a certain time.

### CCK-8

The cell viability was detected by CCK-8 assay. In brief, after BC cell incubating with diverse treatments for 24, 48, and 72 h, cells seeded in 96-well plates were added with 10% CCK-8 reagent (Dojindo) and cultivated for another 2 h, followed by being subjected to detecting the optical density (OD) of each well at 450 nm with a microplate reader (Bio-Rad, Hercules, CA, USA).

### Edu staining

According to protocols provided by the manufacturer, EdU Staining Proliferation Kit was utilized to assess the proliferation of cells after finishing different treatments. By using a BX51 microscope, images were obtained to observe and calculate EdU-positive cells.

### Wound healing

To evaluate the cell migration, a density of 2 × 10^5^ cells/well was seeded into six-well plates. The 200-µL pipette tip was utilized to create a similar size of scratches after a monolayer of cells had formed. Then, scratched cells were removed by three gentle rinses of PBS; the remaining cells were given diverse treatments and cultured in DMEM without FBS for 24 h. A BX51 microscope was utilized to photograph the same position of scratches at 0 and 24 h after scratching.

### Transwell

The invasive ability of BC cells was also evaluated by using Transwell chambers pre-coated with Matrigel. Briefly, BC cells were seeded into the upper chambers containing 200 µL DMEM with or without *F. nucleatum* or Fn-EVs. Simultaneously, 700 µL DMEM with serum was added to the lower chamber. After 24 h cultivation, cells still in the upper chamber were removed, while cells traversing the membranes to the lower chamber were fixed in 4% PFA and stained with 0.1% crystal violet for 15 min. Under a BX51 microscope, the stained cells were photographed and counted in six random visual fields.

### Western blot

BC cells from different groups were lysed with RIPA Buffer on ice to obtain the total protein. After determining the concentration of total protein by the BCA method, the quantitated protein was separated on SDS-PAGE gels and subsequently transferred onto PVDF membranes. Membranes were blocked with skimmed milk, followed by incubation with anti-TLR4 or anti-GAPDH antibodies. Next, the membranes were rinsed thrice with TBST prior to incubation with the secondary antibody. Finally, by using an ECL kit, the protein bands were visualized, of which intensities were measured by Image J. The antibodies information was presented in Supplementary Table S1.

### RT-qPCR

By using Rneasy reagents (Qiagen), total RNA was prepared from tissues or cells. After the quantification and integrity analysis of total RNA, 1 µg total RNA was subjected to reverse transcription using a Reverse Transcription System Kit (Promega) to obtain cDNA. Using an SYBR Premix EX Taq™ II kit (Takara), qRT-PCR was carried out on a Bio-Rad system to detect the expression of TLR4. The relative expression of TLR4 was calculated with GAPDH as the internal control based on the 2^−ΔΔCT^ method [28]. Primer sequences were presented in Supplementary Table S2.

### Cell transfection

Short hairpin (sh) RNA against TLR4 (sh-TLR4#1, sh-TLR4#2, and sh-TLR4#3:) and the scrambled negative control (sh-NC) were purchased from Genepharma (China). The shRNA sequences were presented in Supplementary Table S2. When cell confluency was about 60%, MDA-MB-231 cells were transfected with sh-TLR4#1/2/3 or sh-NC for 48 h using Lipofectamine 2000 (Invitrogen). After transfection finished, cells were harvested and transfection efficiency was analyzed.

### Animal experiments

All procedures regarding animals in this study were approved by the Institutional Animal Care and Use Committee of Huazhong University of Science and Technology Union Shenzhen Hospital and the Affiliated Shenzhen Sixth Hospital of Shenzhen University. All methods were carried out in accordance with relevant guidelines and regulations and ARRIVE guidelines (https://arriveguidelines.org) for the reporting of animal experiments. Before the beginning of in vivo study, thirty-six BALB/c nude mice (female, 6–8 weeks, 18-22 g) purchased from Guangdong medical laboratory animal center were subjected to one-week acclimation. MDA-MB-231 cells were stably transfected with either sh-NC or sh-TLR4 lentivirus before being subcutaneously injected into nude mice.

In brief, mice were randomly divided into three groups (n = 6/group): LV-sh-NC + PBS, LV-sh-NC + Fn-EVs, and LV-sh-TLR4 + Fn-EVs groups. On day one, 1 × 10^6^ sh-NC transfected MDA-MB-231 cells (the former two groups) or TLR4-silencing MDA-MB-231 cells were subcutaneously injected into the inguinal mammary fat pads of mice. One week later, mice were given intratumorally PBS or Fn-EVs (100 µg/mL) for two weeks. Tumor formation was monitored beginning at day 7 every three days, and the growth curve of tumors (volume = (L × W^2^)/2, where W represents the width, and L represents the length) was plotted. One day 28, mice were sacrificed by cervical dislocation, and tumors were therefore harvested and weighed.

For the study of BC metastasis to the liver, the groups defined as above-mentioned, MDA-MB-231 cells were injected into the portal vein of the liver of mice on day one. One week later, mice were given PBS or Fn-EVs (100 µg) via the tail vein for two weeks. After finishing the treatment (day 28), liver tissues were dissected and subjected to H&E staining to investigate liver metastasis.

### Statistics

Pearson’s chi-squared test was applied to analyze the associations between *F. nucleatum* abundance and patient clinical characteristics. Data were represented as the mean ± SD and analyzed using the student t-test or one-way analysis of variance followed by Tukey’s post hoc test on GraphPad Prism software. *P < 0.05* means that the difference is significant.

## Results

### ***F. nucleatum*** was overabundant in BC

**Fig. 1:**
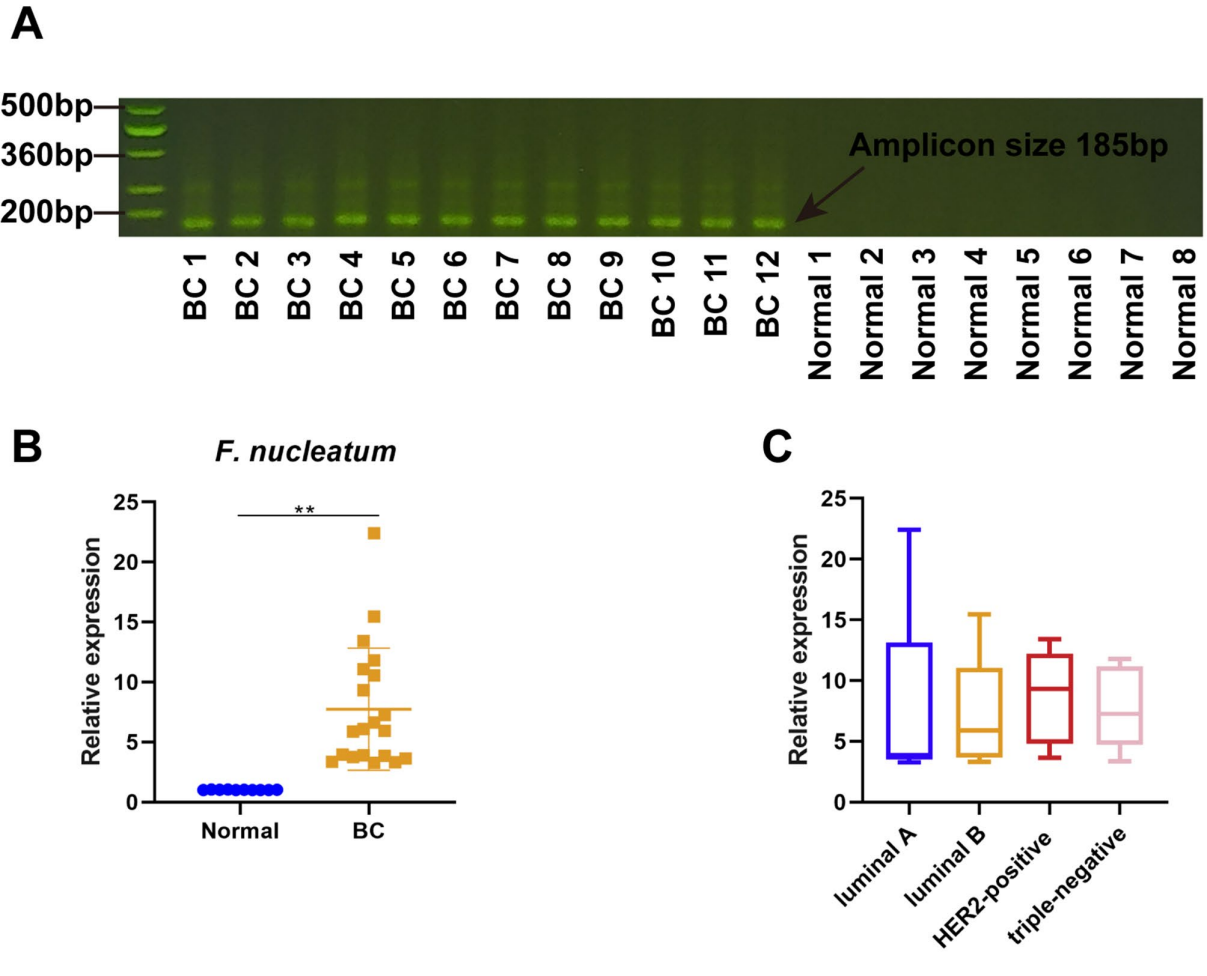
***F. nucleatum *is overabundant in BC. A**. The Northern blot of *F. nucleatum* in normal (n = 8) and cancerous (n = 12) breast tissues **B**. The expression levels of *F. nucleatum* in normal (n = 10) and cancerous (n = 20) breast tissues were detected by RT-qPCR. **C**. The expression levels of *F. nucleatum* in cancerous breast tissues from luminal A (n = 5), luminal B (n = 5), HER2-positive (n = 5), and triple-negative (n = 5) BC patients. Data were analyzed using the student t-test or one-way analysis of variance followed by Tukey’s post hoc test; ^**^p < 0.01

Initially, our study observed whether *F. nucleatum* occurred in BC, and if so, whether its presence correlates with the clinical characteristics of BC patients. Compared with normal breast tissues, *F. nucleatum* was overabundant in BC tissues (Fig. 1A and B). However, there was no significant difference in the *F. nucleatum* levels of different subtypes of BC patients (Fig. 1C). More importantly, our results showed that *F. nucleatum* gDNA expression levels were significantly correlated with both tumor size (*P = 0.0029322*) and metastasis (*P = 0.0403909*) (Table 1), which let us suppose that *F. nucleatum* maybe contribute to the tumor growth and metastasis in BC.

**Table 1:** Association between *F. nucleatum* gDNA expression and clinical characteristics

	All patients	*F. nucleatum* gDNA level		p-value*
		Low expression	High expression	
Total number	20	8	12	
Age (years)				0.8521789
< 60	12	5	7	
≥ 60	8	3	5	
Median (range)	57 (37–77)			
Tumor size (cm^3^)				0.0029322
< 5	14	7	7	
≥ 5	6	1	5	
Pathological grade				0.2918405
I–II	15	5	10	
III-IV	5	3	2	
Metastasis				0.0403909
Yes	8	1	7	
No	12	7	5	

### Fn-EVs enhanced BC cell proliferation, migration, and invasion

**Fig. 2:**
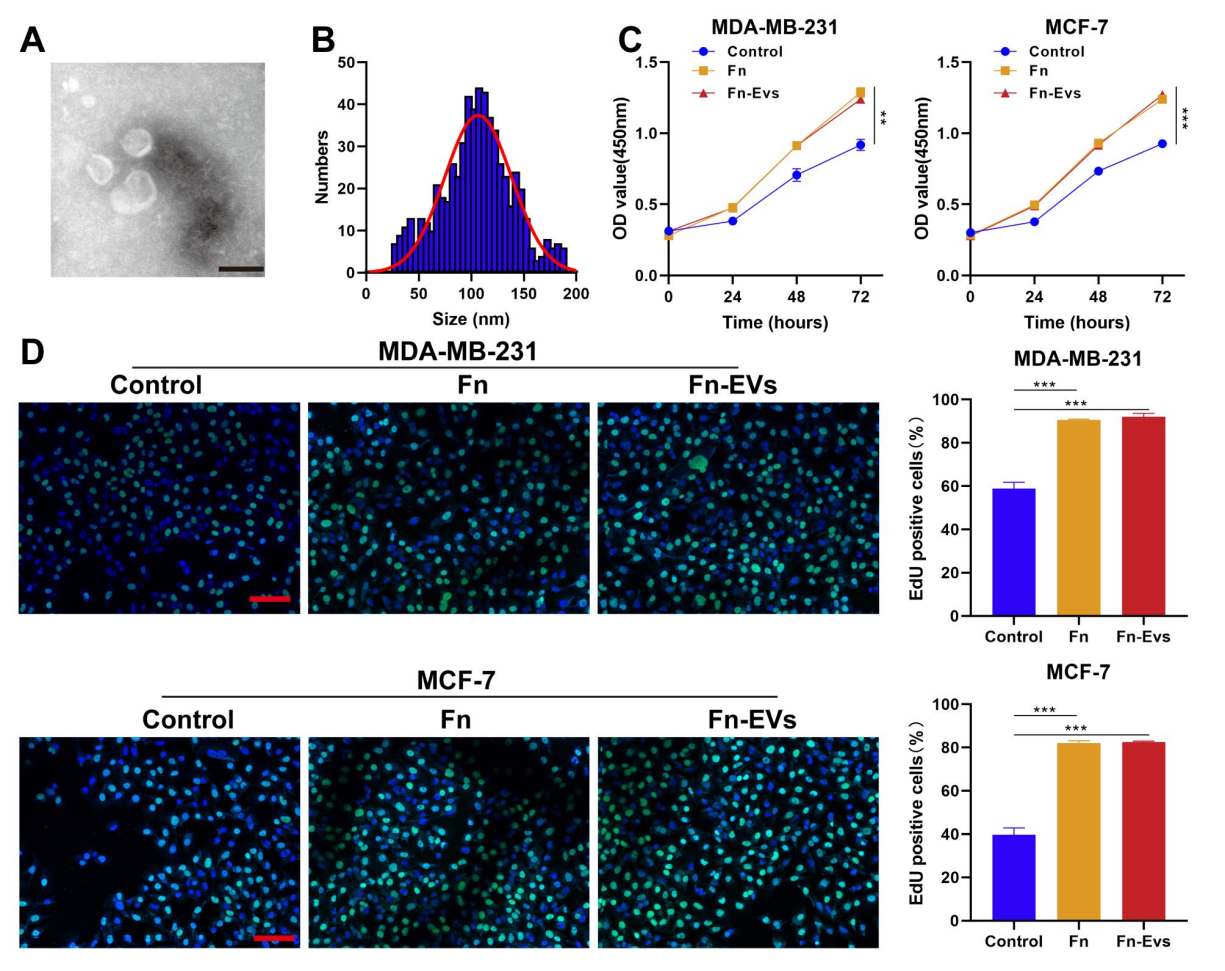
***F. nucleatum *-derived EVs enhanced BC cell viability and proliferation A**. Morphology of the isolated *F. nucleatum*-derived EVs was observed under TEM. (Scalebar, 100 nm) **B**. Size distribution of Fn-EVs was analyzed by NTA. MDA-MB-231 and MCF-7cells were treated with PBS, *F. nucleatum*, or Fn-EVs (n = 3 per group) **C**. The cell viability was detected by CCK-8 after diverse treatments for 24, 48, and 72 h **D**. The cell proliferation was detected by Edu staining after 24 h (Scalebar, 100 μm). Data were analyzed using the one-way analysis of variance followed by Tukey’s post hoc test; ^**^p < 0.01, ^***^p < 0.001

To study the role of Fn-EVs in the malignant phenotypes of BC, Fn-EVs were isolated from the culture supernatants of *F. nucleatum* (ATCC 25,586) via a centrifugal ultrafiltration-based method. TEM was exploited to observe the morphology of the isolated Fn-EVs, which showed Fn-EVs with a round or oval membrane (Fig. 2A). Additionally, NTA revealed that the diameter of the Fn-EVs ranged from 20 to 190 nm of which peak diameters were approximately 100 nm (Fig. B). These results collectively suggested the successful isolation of Fn-EVs.

**Fig. 3:**
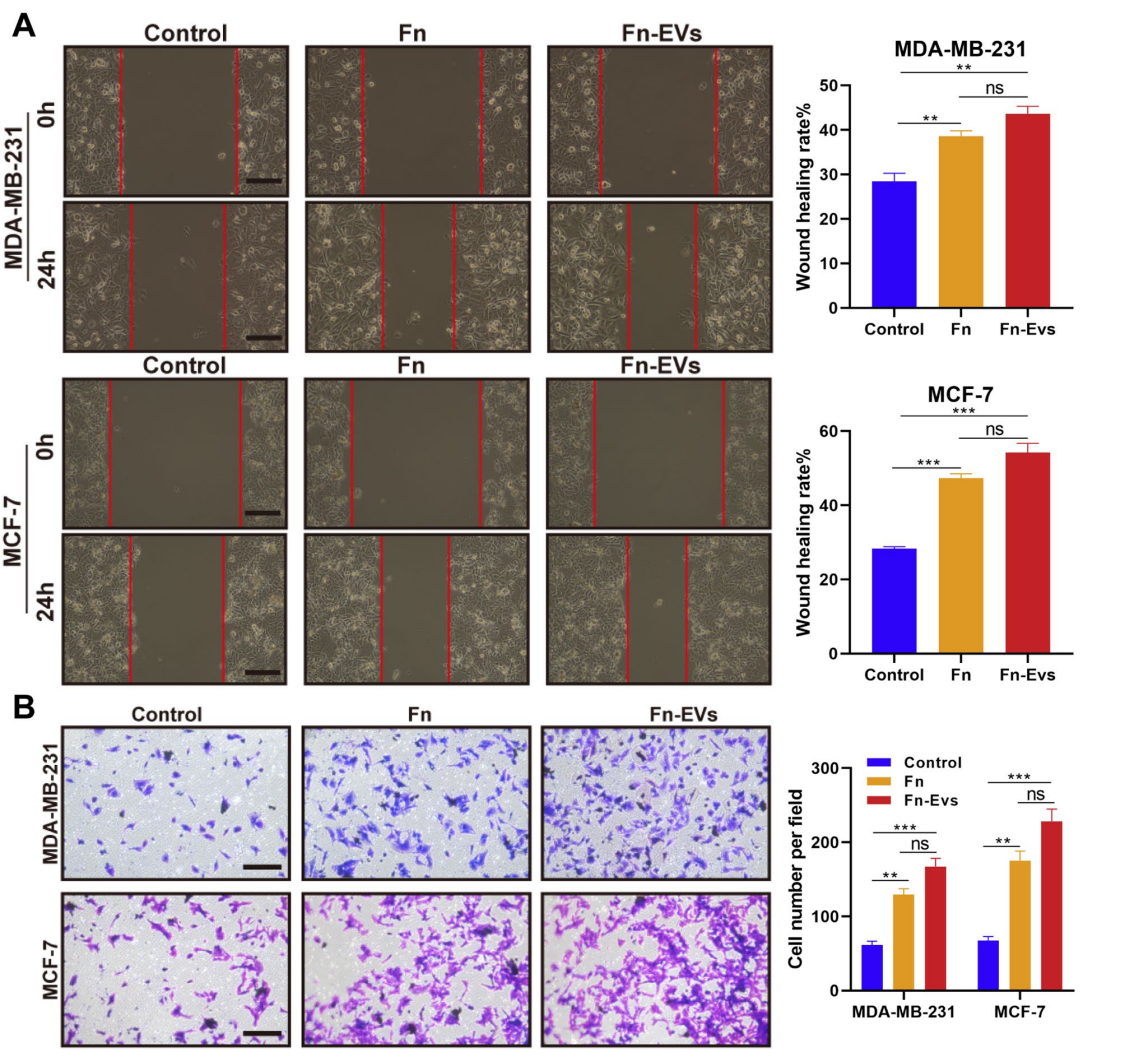
*** F. nucleatum*-derived EVs facilitated the migration and invasion of BC cells**. MDA-MB-231 and MCF-7cells were treated with PBS, *F. nucleatum*, or Fn-EVs for 24 h (n = 3 per group) **A**. The cell migration was detected by wound healing assay (Scalebar, 100 μm) **B**. The cell invasion was observed by Transwell assay (Scalebar, 100 μm). Data were analyzed using the one-way analysis of variance followed by Tukey’s post hoc test; ^**^p < 0.01, ^***^p < 0.001

Next, two BC cell lines (MDA-MB-231 and MCF-7) were respectively incubated with *F. nucleatum* (defined as the Fn group) and Fn-EVs to investigate how EVs released by *F. nucleatum* influence various cancer hallmarks of recipient cells. After diverse treatments for 24, 48, and 72 h, the cell viability of both the Fn and Fn-EVs groups was significantly higher than that of the control group (cells treated with PBS) (Fig. 2C). Edu staining revealed that exposing BC cells to *F. nucleatum* or Fn-EVs led to a significant increase in their proliferative ability (Fig. 2D). Besides, both the migration and invasion of BC cells were also markedly increased after the administration of *F. nucleatum* or Fn-EVs (Fig. 3A and B). The effects of Fn-EVs on BC cell migration and invasion were slightly stronger than those of *F. nucleatum*, but there was no significance (Fig. 3A and B). Collectively, Fn-EVs could strengthen the malignant behaviors of BC cells, which included proliferation, migration, and invasion.

### Fn-EVs contribute to BC cell proliferation, migration, and invasion via activation of TLR4

**Fig. 4:**
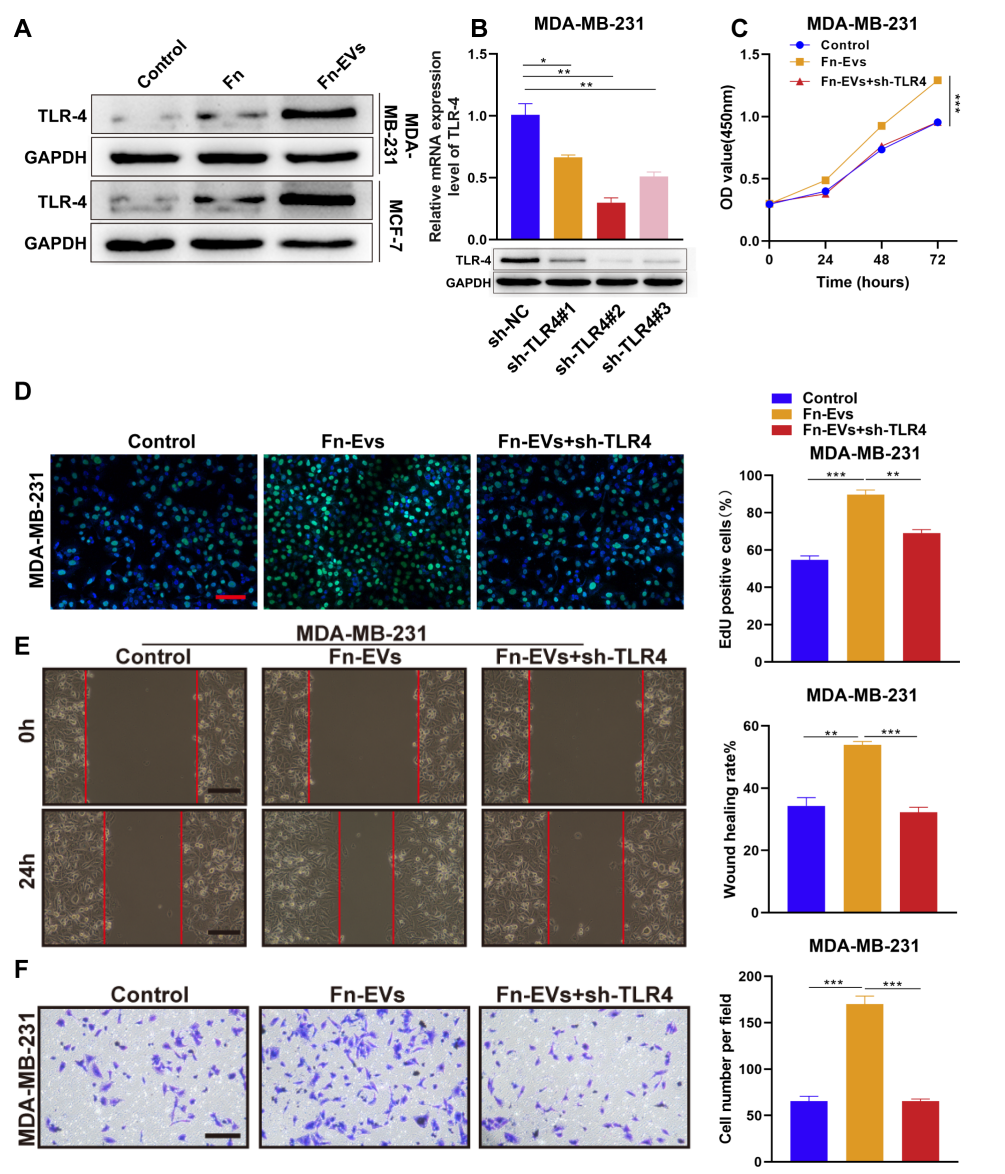
*** F. nucleatum*-derived EVs contribute to BC cell proliferation, migration, and invasion via activation of TLR4 A**. The expression of TLR4 protein in BC cells with different treatments was examined by western blot (n = 3 per group) **B**. The transfection efficiency of sh-TLR4#1, #2, and #3 was determined by (top) RT-qPCR and (bottom) western blot (n = 3 per group). MDA-MB-231 cells were treated with PBS, Fn-EVs, or sh-TLR4 transfection combined with Fn-EVs (n = 3 per group) **C**. The cell viability was detected by CCK-8 after diverse treatments for 24, 48, and 72 h **D**. The cell proliferation was detected by Edu staining after 24 h (Scalebar, 100 μm) **E**. The cell migration was detected by wound healing assay after 24 h (Scalebar, 100 μm) **F**. The cell invasion was observed by Transwell assay after 24 h (Scalebar, 100 μm). Data were analyzed using the one-way analysis of variance followed by Tukey’s post hoc test; ^*^p < 0.05, ^**^p < 0.01, ^***^p < 0.001

Given that *F. nucleatum* has been reported to promote tumorigenesis through TLR4 signaling, we wonder whether Fn-EVs facilitate the malignant phenotypes of BC cells via TLR4 as well. As expected, the treatment of both *F. nucleatum* and Fn-EVs could increase TLR4 protein expression of BC cells (Fig. 4A). Notably, Fn-EVs caused an obvious elevation in TLR4 levels. To verify whether the effects of Fn-EVs on BC cells rely on TLR4 activation, we further explore the role of Fn-EVs in MDA-MB-231 cells after knocking down TLR4. RT-qPCR and western blot confirmed that the transfections of sh-TLR4#1, #2, and #3, all could effectively silence TLR4 expression in MDA-MB-231 cells, while sh-TLR4#2 exhibited the strongest efficacy (Fig. 4B). Hence, sh-TLR4#2 was chosen for the following experiments. Our results showed that Fn-EVs-induced promotion in the proliferation, migration, and invasion of MDA-MB-231 cells was abolished by the transfection of sh-TLR4 (Fig. 4C-F). Thus, the promotive effects of Fn-EVs on the proliferation, migration, and invasion of BC cells may be attributed to the activation of the TLR4 pathway by Fn-EVs.

### Fn-EVs enhanced tumor growth and metastasis via activation of TLR4 in BC

**Fig. 5:**
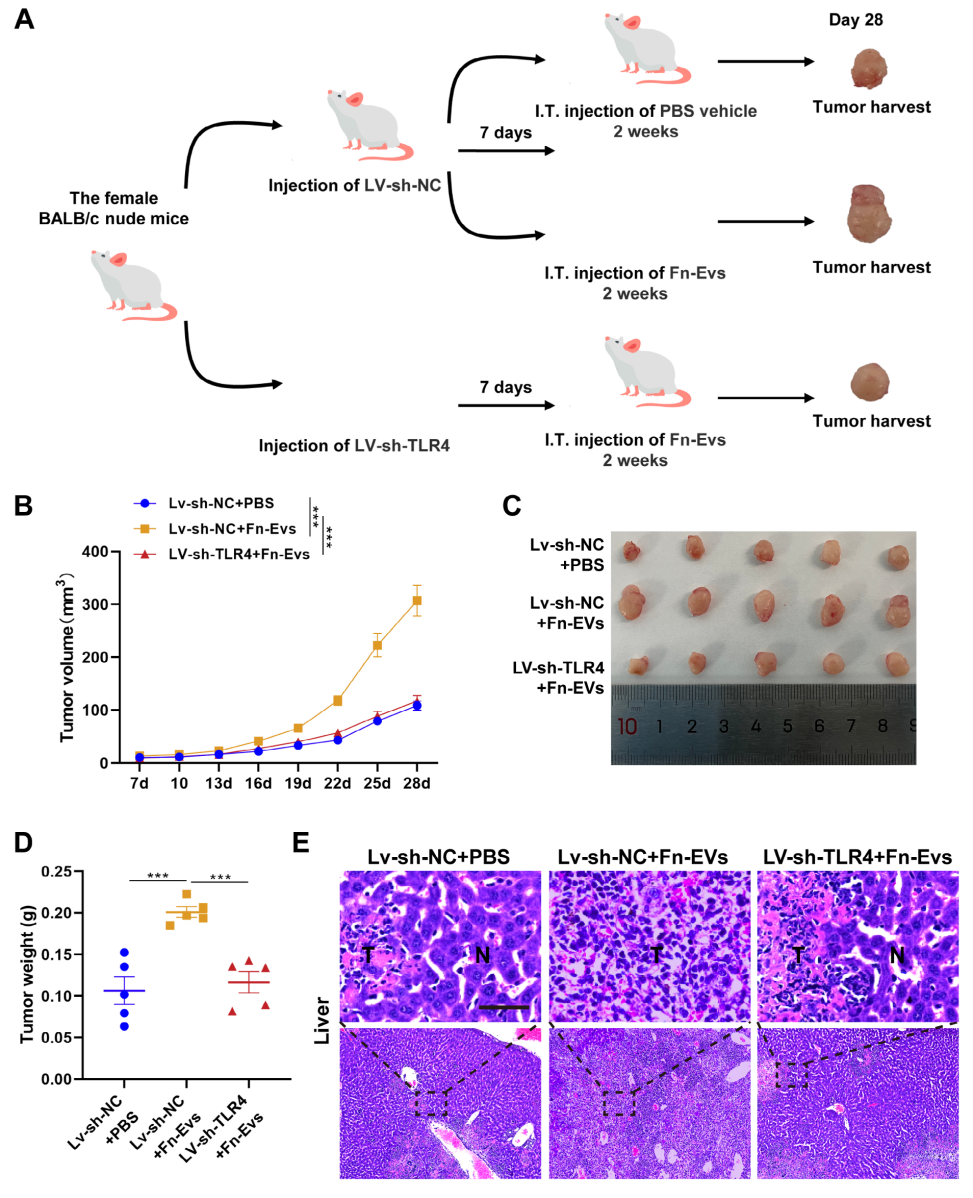
***F. nucleatum*-derived EVs enhanced tumor growth and liver metastasis via activation of TLR4 in BC A**. Experimental design of in vivo study BALB/c nude mice were randomly divided into three groups (n = 6/group): LV-sh-NC + PBS, LV-sh-NC + Fn-EVs, and LV-sh-TLR4 + Fn-EVs groups. One-week later cell inoculation, mice were given intratumorally or intravenously PBS or Fn-EVs (100 µg/mL) for two weeks **B**. Tumor growth curve **C**. The tumor harvested from each group on day 28 **D**. Weight of the harvested tumor **E**. H&E staining for the liver tissues from the metastatic murine model (Scalebar, 100 μm). Data were analyzed using the one-way analysis of variance followed by Tukey’s post hoc test; ^***^p < 0.001

Finally, BALB/c nude mice xenograft model was established to support the in vitro studies. The animal experimental design was described as Fig. 5A. The tumor growth curve showed that the tumor growth of nude mice treated with Fn-EVs was significantly faster than those treated with PBS (Fig. 5B). The tumor images and tumor weight on day 28 further indicated that Fn-EVs exerted a significant promotion effect on the growth of subcutaneous tumors (Fig. 5C and D). Next, MDA-MB-231 cell suspension was injected into the portal vein of the liver of mice to evaluate the function of Fn-EVs on BC liver metastasis. H&E staining results showed that liver sections in the Fn-Ex group exhibit the highest number and largest area of cancerous nests in the lung (Fig. 5E). To confirm that Fn-EVs promotes BC progression through TLR4, the xenograft model established by TLR4-silencing MDA-MB-231 cells inoculation. Notably, Fn-EVs-enhanced tumor growth and liver metastasis were rescued by TLR4 silence (Fig. 5B-E). Collectively, coincident with the results of in vitro experiments, Fn-EVs significantly facilitate tumor growth and metastasis through the TLR4 pathway in BC.

## Discussion

As a community of microorganisms, the microbiota exhibits homeostatic properties and can modulate immunity [30], which is found in all multicellular organisms, including humans. In the past few decades, the importance of microbiota in human health and disease, such as cancer, has been increasingly emphasized as its involvement in regulating physiological processes [31]. A growing number of studies revealed that an elevated or reduced risk of certain cancers is attributed to colonization with various micro-organisms [18, 32]. Hence, identifying the specific oncogenic micro-organisms involved and the mechanism of their effect could be beneficial in the management of the onset and development of tumors. A recent study demonstrated that different tumor types, such as glioma, pancreatic cancer, and BC, have distinct microbial compositions [33]. Notably, among multiple cancers, a particularly rich and diverse microbiome has been observed in BC [33]. Wilkie et al. revealed that BC commensal microbiota is distinctly composed of Gram-negative bacteria and highlighted the role of bacterial components in BC development [34]. As one of the recently identified BC-related microbes, *F. nucleatum* has gained the most attention. The responsibility of *F. nucleatum* in BC development and metastatic progression was identified [18]. Besides, it has been reported that *F. nucleatum* might accelerate cell growth and treatment resistance in BC by activating autophagy and immune evasion by suppressing the immune system [35]. Consistent with the previous publication, our research showed that the *F. nucleatum* is overabundant in BC tissues. The high *F. nucleatum* levels were positively correlated with the tumor size and whether the metastasis occurred, suggesting that *F. nucleatum* maybe contribute to the worse clinical outcomes.

To date, multiple research projects have supported the causal role of EVs in cancer progression [36]. However, almost all of these reports have focused on the EVs isolated from various types of cells, but few have focused on the bacterial EVs. In recent years, EVs derived from bacteria have been proven to play important roles in bacteria–host interactions during disease progression [37]. In the present study, our study reveals that *F. nucleatum*-derived EVs play a newly identified role in promoting tumor development. For the in vitro experiments on two BC cell lines, we found that *F. nucleatum*-derived EVs exhibited a similar effect to *F. nucleatum* on BC cell viability and proliferation, revealing the promotion effect of *F. nucleatum*-derived EVs on tumor growth in BC, which was corroborated by the further in vivo study. Metastasis leads to more than 90% of cancer-associated mortality in general, and is mainly responsible for the death of BC. The 5-year survival rate of patients with localized BC is almost 98%, while that of patients with metastatic BC drops to 26% [38]. Interestingly, the analyses of cell migration and invasion also exhibited a similar tendency to those of cell proliferation. In addition, the analysis of a murine model of liver metastasis indicated a potent function of *F. nucleatum*-derived EVs on BC liver metastasis.

Several groups have previously characterized mechanisms underlying the promotive effect of *F. nucleatum* in colorectal cancer (CRC) progression [39]. The function of *F. nucleatum* on the chemoresistance of CRC is associated with its modulation of autophagy [40]. Another study reported that *F. nucleatum* suppresses adaptive immunity mediated by antitumor T-cells in CRC by regulating the expression of microRNA [41]. More importantly, increasing studies demonstrated that *F. nucleatum* contributes to the change of tumor microenvironment and the progression of CRC via a TLR4-dependent mechanism [11, 42, 43]. Significance regarding TLR4 activation in BC progression has been reported in a large number of studies [44, 45]. For example, a previous study demonstrated that pharmacologic TLR4 inhibition could suppress the tumor growth of TP53 mutant BC [46]. Hence, we speculated that *F. nucleatum* and its derived EVs might facilitate BC progression through similar mechanisms. Our data indicated that both *F. nucleatum* and *F. nucleatum*-derived EVs resulted in a significant increase in the TLR4 expression, while *F. nucleatum*-derived EVs exerted a stronger effect. Importantly, in vitro and in vivo studies collectively confirmed that *F. nucleatum*-derived EVs contribute to tumor growth and liver metastasis in BC through a TLR4-dependent mechanism since the knocking down of TLR4 in BC cells effectively counteracted the effect of *F. nucleatum*-derived EVs. In summary, the current study first revealed the role of *F. nucleatum*-derived EVs in BC progression and demonstrated the mechanism is related to TLR4 activation.

However, there are several limitations in the present study. First, the main gap is whether Fn-EVs can induce normal breast cells to become malignant remains unclear, and the normal breast cell lines would be used to validate our findings in subsequent experiments. In addition, the mechanism exploration in this study is rudimentary, how TLR4 was activated by *F. nucleatum*-derived EVs should also be further researched. Lastly, the lack of an orthotopic tumor model for in vivo experiments minimizes the reliability of our findings; therefore, the role and the specific mechanism of *F. nucleatum*-derived EVs in BC growth and metastasis should be further verified in an orthotopic tumor model in the future.

## Conclusions

In conclusion, our present study authenticated that the *F. nucleatum*-derived EVs manifested a promotive function on tumor growth and metastasis of BC, which might be associated with its role in TLR4 activation. This finding provides a novel insight into the role of bacterial EVs in cancer development and some scientific information on the interaction between microbiome and tumor.

## Electronic supplementary material

Below is the link to the electronic supplementary material.

Supplementary Material 1

Supplementary Material 2

Supplementary Material 3

Supplementary Material 4

## Data Availability

The datasets used and/or analysed during the current study are available from the corresponding author on reasonable request.

## List of Abbreviations

*F. Nucleatum*: *Fusobacterium nucleatum*
BC: Breast cancer
CRC: Colorectal cancer
Fn-EVs: *Fusobacterium nucleatum*-derived small extracellular vesicles
NTA: Nanoparticle tracking analysis
OD: Optical density
TEM: Transmission electron microscope
TLR4: Toll-like receptor 4
