# Supplementary material for: Fusobacterium nucleatum-derived small extracellular vesicles facilitate tumor growth and metastasis via TLR4 in breast cancer
Source: BMC Cancer. 2023 May 23;23:473. doi: 10.1186/s12885-023-10844-z (PMC10207721; doi:10.1186/s12885-023-10844-z)
Supplement: Supplementary file 1 — Supplementary Material 1 [file 12885_2023_10844_MOESM1_ESM.docx]

**Supplementary Table S1. Primer sequences**

| **Gene** | **Sequence** |  |
| --- | --- | --- |
| *F. nucleatum* ATCC 237726  nusG | Forward | CAACCATTACTTTAACTCTACCATGTTCA |
|  | Reverse | ATTGACTTTACTGAGGGAGATTATGTAААААТC |
| TLR-4 | Forward | AGACCTGTCCCTGAACCCTAT |
|  | Reverse | CGATGGACTTCTAAACCAGCCA |
| GAPDH | Forward | ACCACAGTCCATGCCATCAC |
|  | Reverse | CAGCTCAGGGATGACCTTGC |
| sh-TLR4#1 |  | AGACTACTACCTCGATGATAT |
| sh-TLR4#2 |  | CCGCTGGTGTATCTTTGAATA |
| sh-TLR4#3 |  | GCCACCTCTCTACCTTAATAT |
