# Supplementary material for: Fusobacterium nucleatum-derived small extracellular vesicles facilitate tumor growth and metastasis via TLR4 in breast cancer
Source: BMC Cancer. 2023 May 23;23:473. doi: 10.1186/s12885-023-10844-z (PMC10207721; doi:10.1186/s12885-023-10844-z)
Supplement: Supplementary file 2 — Supplementary Material 2 [file 12885_2023_10844_MOESM2_ESM.docx]

**Supplementary Table S2. The antibodies used in this research**

| **Antibody** | **Manufacturer** | **Cat.no** |
| --- | --- | --- |
| TLR4 | Abcam | ab13556 |
| GAPDH | Abcam | ab9484 |
