# Supplementary figures and images for: Fusobacterium nucleatum-derived small extracellular vesicles facilitate tumor growth and metastasis via TLR4 in breast cancer
Source: BMC Cancer. 2023 May 23;23:473. doi: 10.1186/s12885-023-10844-z (PMC10207721; doi:10.1186/s12885-023-10844-z)

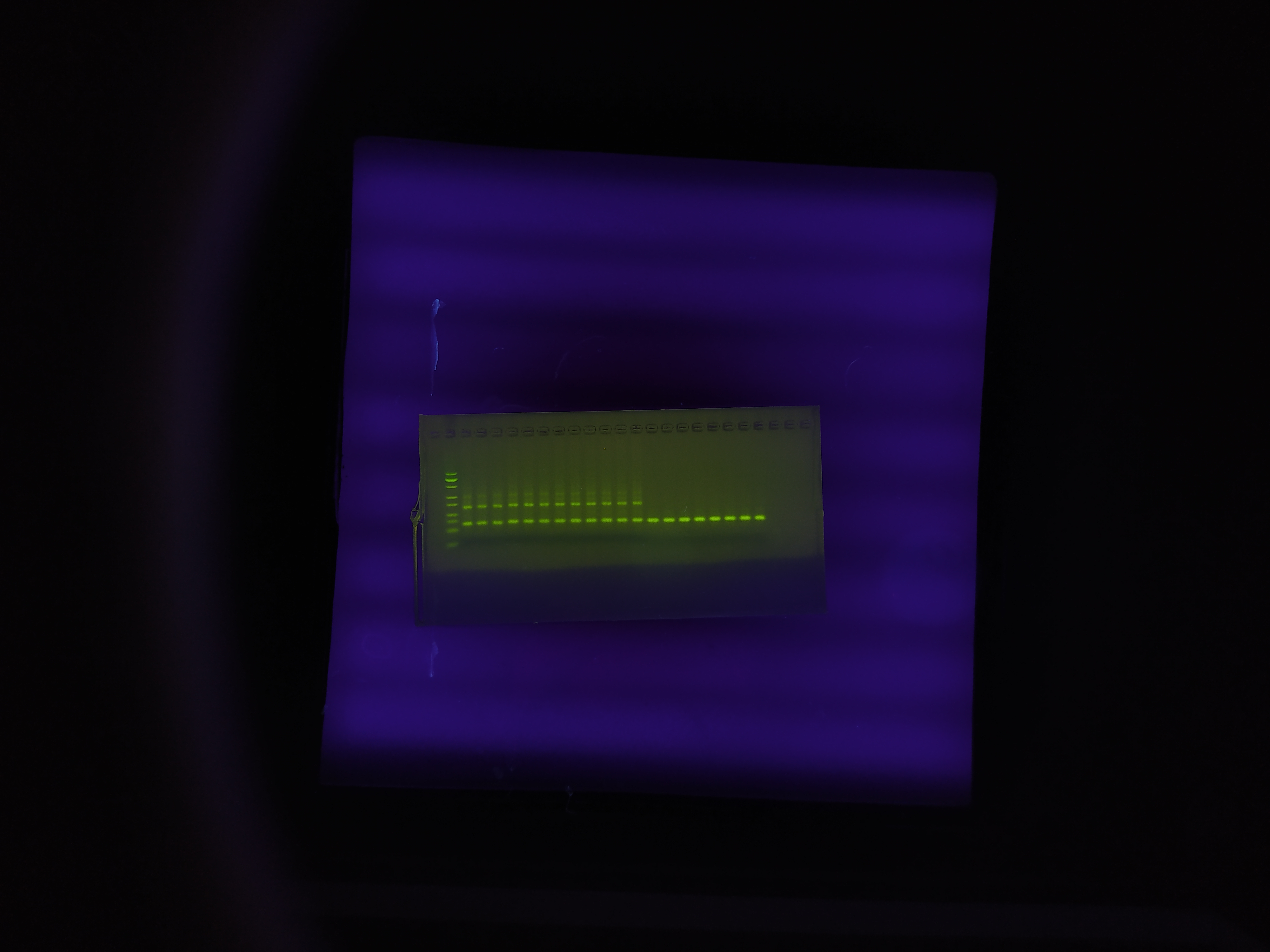

Supplement: Supplementary file 3 — Supplementary Material 3 [file 12885_2023_10844_MOESM3_ESM.jpg]

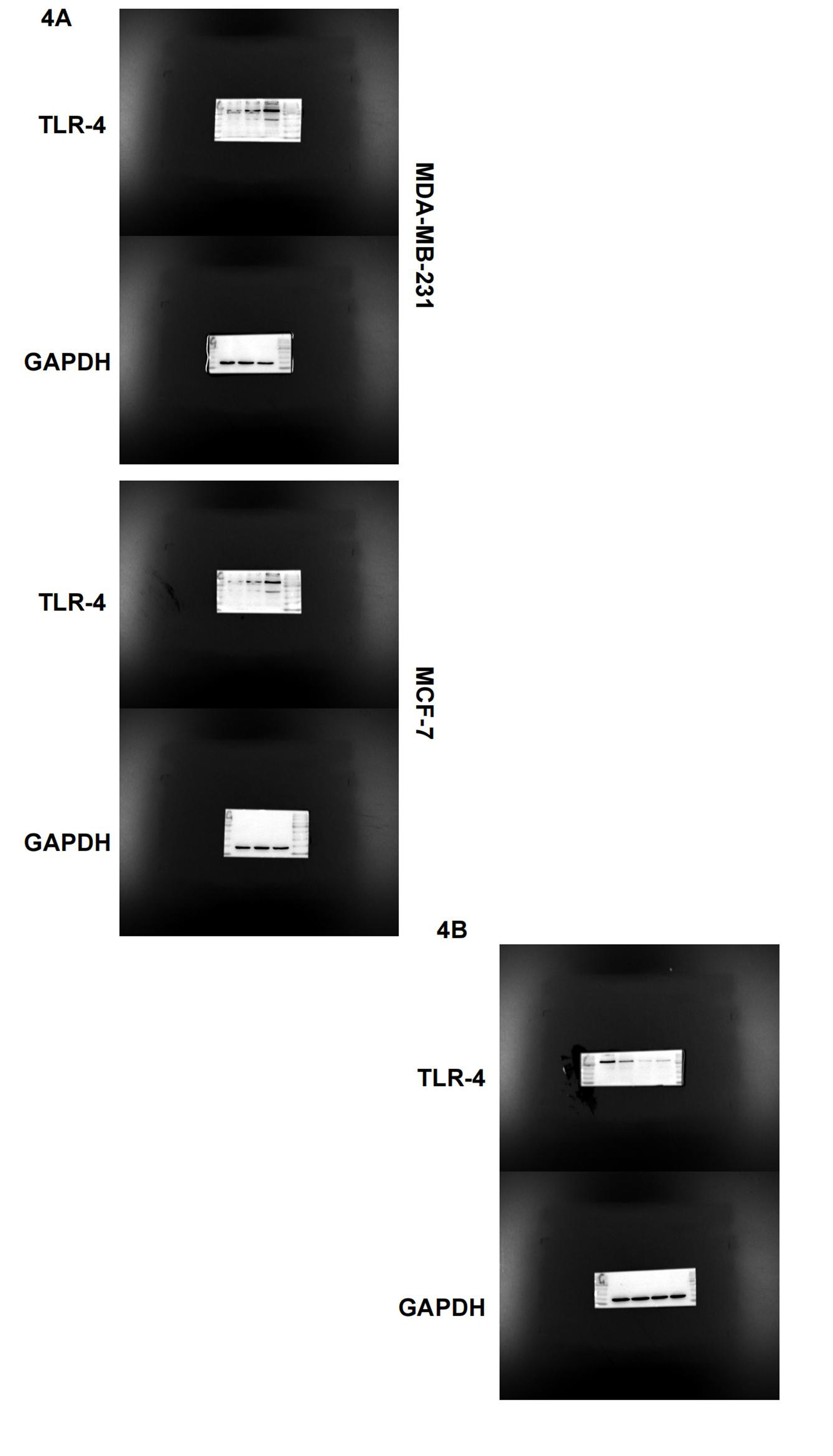

Supplement: Supplementary file 4 — Supplementary Material 4 [file 12885_2023_10844_MOESM4_ESM.jpg]
